# Auricular Acupuncture for Facial Aesthetics: A Preliminary Retrospective Clinical Study of 217 Cases

**DOI:** 10.1111/jocd.70629

**Published:** 2026-01-07

**Authors:** Wangpiaoyun Zhu, Panita Prateepjumraskul, Yike Han, Hantong Hu, Lifang Chen

**Affiliations:** ^1^ The Third Clinical Medical College of Zhejiang Chinese Medical University Hangzhou Zhejiang Province China; ^2^ International Education College of Zhejiang Chinese Medical University Hangzhou Zhejiang Province China; ^3^ Department of Acupuncture The Third Affiliated Hospital of Zhejiang Chinese Medical University Hangzhou Zhejiang Province China

**Keywords:** acupuncture cosmetology, auricular acupuncture, medical cosmetology, retrospective study

## Abstract

**Objective:**

In recent years, acupuncture has gained attention as a safe and natural method in cosmetic medicine. Auricular acupuncture has been insufficiently studied in the field of facial aesthetics. Our previous clinical observations were associated with rapid facial tightening, lifting, and slimming following auricular acupuncture, which is characterized by its simplicity, safety, and convenience.

**Methods:**

In this retrospective study, we collected data from 242 participants who received auricular acupuncture for facial cosmetic treatment. Participants received weekly treatments, with facial photographs taken before and after each session. Outcome measures included the Wrinkle Severity Rating Scale (WSRS), Global Aesthetic Improvement Scale (GAIS), Visual Analogue Scale (VAS), and FACE‐Q score. Evaluations were performed at three time points: before the first treatment, immediately after the first treatment, and following 3 months of continuous treatment. The safety assessment monitored adverse reactions during the trial period.

**Results:**

A total of 217 participants were included in the statistical analysis as valid cases, of which 131 were assessed for immediate response and 86 for intermediate‐term response. Immediate results showed a statistically significant decrease in WSRS scores (from 2.49 ± 0.69 to 1.86 ± 0.87, *p* < 0.05) and a 77.10% improvement in GAIS. Efficacy observation at 3 months showed WSRS score decreased from 2.53 ± 0.66 to 1.67 ± 0.73 (*p* < 0.05) and GAIS improvement of 95.35%. Participants reported high overall satisfaction with their facial appearance. After immediate treatment, the improvement in nasolabial folds was the most effective, with a score of 3.59 ± 0.62 (out of 5.00); after intermediate‐term treatment, the improvement of the cheeks was the most effective, with a score of 3.78 ± 0.64 (out of 5.00). In both immediate and intermediate‐term treatments, improvements were observed in the lower face and jawline, with relatively high satisfaction. Minimal pain was noted, with 64.98% reporting slight discomfort and 35.02% reporting no pain. No severe events were observed, affirming the treatment's safety and comfort.

**Conclusions:**

Auricular acupuncture was associated with improvements in both immediate and intermediate‐term evaluations. The immediate effects include facial slimming, lifting, reduction of nasolabial folds, and improvement of nasolabial lines. Intermediate‐term effects include tightening of the face, promotion of facial rejuvenation, overall facial enhancement, and an increase in participant satisfaction. Nonetheless, in view of multiple inherent limitations as a retrospective study, the findings should be regarded as preliminary and interpreted with caution, and future studies should employ prospective, adequately powered multicenter designs with longer follow‐up, validated patient‐reported outcomes, standardized image acquisition, and integration of objective imaging and biomechanical metrics.

## 1 Introduction

Currently, non‐surgical aesthetic procedures are gaining popularity, reflecting the rising demand for minimally invasive, highly safe, natural, and healthy medical beauty techniques [1]. As a health‐focused and minimally invasive approach to medical aesthetics, acupuncture aligns with this trend, which has contributed to its increasing popularity [2]. In recent years, studies suggest that acupuncture, as an alternative therapy, may improve skin condition, relieve wrinkles, and attenuate signs of skin aging [3, 4].

There are various treatment methods in cosmetic acupuncture, such as facial needling, moxibustion, cupping therapy, scraping, acupoint thread embedding, and auricular therapy [5]. Auricular acupuncture, a type of acupuncture that involves inserting needles into specific points on the auricle, stands out among the various treatment options due to its indirect application to the face, making it a potentially attractive option in aesthetic practice [6].

In China, the *Yellow Emperor's Internal Classic*, dating back about 3000 years, records associations between the auricle and human health as well as disease prevention and treatment [7]. Outside China, French physician Paul Nogier in 1957 proposed the “inverted fetus” hypothesis following systematic investigations, positing a somatotopic mapping of body regions onto the external ear [7]. In 1958, Nogier's auricular map was introduced to China, after which auriculotherapy has developed continuously and is now widely used in outpatient and inpatient care as well as home practice [8]. In outpatient care, some practitioners use auriculotherapy alone, which is generally well accepted by patients. Clinically, auriculotherapy is applied across multiple areas; currently, most research focuses on neuropsychiatric conditions including sleep disorders, pain syndromes and musculoskeletal disorders, internal medicine indications such as gastrointestinal, immunoallergic and otolaryngologic problems, and obstetric care [9, 10, 11, 12, 13, 14]. Treatment methods have also diversified, including auricular massage, auricular seed‐press therapy, auricular acupuncture, auricular moxibustion, auricular thread‐embedding, auricular bloodletting, auricular electroacupuncture [15, 16, 17, 18, 19]. In recent years, clinical interest in using auricular acupuncture for anti‐aging and wrinkle reduction has increased; however, relevant clinical evidence remains limited [20]. Therefore, we conducted an exploratory study to evaluate its cosmetic effects, safety, and patient satisfaction.

## 2 Methods

### 2.1 Study Design and Sample Size

In 2018, we carried out cosmetic acupuncture in clinical practice and accumulated a lot of practical techniques and experience. To standardize the procedures of auricular acupuncture for cosmetic purposes, we regularly held training sessions to provide rigorous instruction for acupuncture practitioners. Additionally, in subsequent clinical treatments, we conducted photographic documentation and scale‐based assessments of patients before and after auricular therapy.

This single‐center, self‐controlled retrospective study was conducted in the outpatient clinic of the Acupuncture Department of a tertiary hospital between November 2023 and December 2024. Participants served as their own controls. For immediate effects, we included individuals who underwent a single session of auricular acupuncture with standardized facial photography immediately before and after the same‐day treatment. For intermediate‐term effects, we included individuals who received weekly auricular acupuncture over approximately 3 months with serial standardized facial photography. Given the retrospective design, we considered all consecutive eligible cases during the study period; the final sample size was determined by the availability of complete, quality‐assured data after applying pre‐specified criteria, and no a priori power calculation was performed.

### 2.2 Ethics Statement

The study complied with the Declaration of Helsinki and institutional guidelines. The Institutional Review Board of The Third Affiliated Hospital of Zhejiang Chinese Medical University deemed this minimal‐risk retrospective analysis exempt from formal ethical review. All participants provided informed consent for clinical photography and use of their data in the study.

### 2.3 Inclusion and Exclusion Criteria

The inclusion criteria were as follows: participants who received cosmetic treatment only with auricular acupuncture, had complete clinical data, including high‐quality, comparable pre‐ and post‐treatment photographs, satisfaction assessments, as well as WSRS and GAIS scores, aged 35–70 years, diagnosed with at least mild nasolabial folds (WSRS score ≥ 2).

The main exclusion criteria were as follows: missing basic information such as age or general health status; poor‐quality photographs, making pre‐ and post‐treatment comparisons impossible; severe systemic comorbidities (e.g., heart failure, liver dysfunction); receiving other facial anti‐aging treatments within the past 6 months; lack of documented informed consent for use of clinical data and photographs in this study.

Participants who met the above criteria received a single session of auricular acupuncture cosmetic treatment, and had standardized photographs taken before and after the treatment were included in the immediate efficacy group. Those who met the criteria completed 3 months of auricular acupuncture cosmetic treatment, and had standardized photographs taken before the first session and after the final session were included in the intermediate‐term efficacy group.

### 2.4 Data and Facial Photograph Collection

Participants' basic information was collected, including name, age, gender, health status, treatment times and frequency, as well as standardized pre‐ and post‐treatment photographs, consent documentation, satisfaction questionnaire, adverse event reports, and other information. To ensure the consistency of facial photographic evidence as much as possible, a standardized protocol was established for all facial image capture. This protocol required a fixed light source, consistent camera distance and angle, and a neutral facial expression from the participant. The data were recorded and compiled by experienced research physicians.

### 2.5 Interventions

#### 2.5.1 Points Selection

Based on the holographic correspondence between auricular points and the whole body, modern auricular medicine [21, 22, 23], and our clinical experience, we adopted a two‐needle protocol involving two insertion points and three anatomically related regions on each ear: (1) a cheek‐related auricular point (“Cheek Area”) on the anterior earlobe, (2) the gonadotropin point at the interauricular notch, and (3) the region overlying the great auricular nerve along the lower posterior ear. Needles were inserted only at the cheek and gonadotropin points; the great auricular nerve region served as the target direction for the cheek needle rather than as a separate insertion point. These two auricular points are experience‐based acupoints derived from prior clinical work by a senior auricular acupuncture expert [24]. All participants were treated using this standardized two‐point protocol, without syndrome differentiation.

#### 2.5.2 Manipulation

The intervention programs were developed according to the Standardized Operation of Acupuncture Techniques—General Rules for Compilation (GB/T 21709.3‐2021). Disposable plastic handle needles with a size of 0.14 × 15 mm were used. Participants were either seated or in a supine position, and all treatments were performed by senior acupuncturists. There is no specific order required for treating the left and right ears.

First, the practitioner sterilized the local skin, located the lowest point of the cheek area on the auricular point, used the thumb and index finger of one hand to fix the earlobe, inserted the needle at a 15‐degree angle to the skin surface to a depth of 0.10–0.30 cm, and advanced it in the direction of the great auricular nerve region until it was fully inserted, without a separate insertion at the great auricular nerve itself, then performed twisting maneuver for approximately 30 s. Next, locate the gonadotropin point and perform the acupuncture operation using the same technique. After completing the acupuncture operation of the above two points, observe whether the facial skin on the same side has achieved tightening effect. Most subjects will show an immediate tightening effect. For participants who do not have an immediate effect, it is recommended to perform twisting manipulation again. Throughout the treatment, participants were not required to experience the sensation of “getting qi” (a feeling of soreness, heavy, or numbness). Some participants might feel mild needling pain, but most consider the discomfort to be mild and well within tolerable limits. The detailed locations of the selected auricular points are shown in Figure S1.

#### 2.5.3 Treatment Frequency and Duration

The needle was retained for at least 2 h, during which the subject should not feel any sensation. Participants were instructed not to touch their ears during needle retention to prevent the needle bending or falling out. After 2 h, the participants were asked to remove the needle by themselves. They were also instructed to prepare a cotton swab in advance and apply gentle pressure to the needle insertion sites for approximately 1 min upon needle removal. Auricular acupuncture was performed once a week, with a continuous treatment period of 3 months.

### 2.6 Outcomes and Definitions

#### 2.6.1 Primary Outcomes

The efficacy of facial rejuvenation was evaluated by WSRS (evaluating the severity of nasolabial folds) and GAIS (Global Aesthetic Improvement Scale).

WSRS [25]: 1–5 ordinal scale (1 = absent, 2 = mild, 3 = moderate, 4 = severe, 5 = extreme; higher = more severe). Change was calculated as ΔWSRS = post–pre; negative values indicate improvement.

GAIS was defined as [26]: 1 = very much improved, 2 = greatly improved, 3 = improved, 4 = no change, and 5 = worse; scores ≤ 3 were prespecified as indicating improvement and also report the full category distribution.

We used an abbreviated set of FACE‐Q domains to assess patient‐reported satisfaction with facial appearance. FACE‐Q is a modular, validated patient‐reported outcome instrument widely used in cosmetic facial outcomes research [27]. Based on early clinical observations suggesting a more pronounced cheek response, we pre‐specified a focused subset of domains: (i) satisfaction with overall facial appearance, (ii) satisfaction with the cheeks, (iii) satisfaction with the lower face and jawline, and (iv) satisfaction with improvement of the nasolabial folds. In line with the modular design of FACE‐Q [28], we selected these validated domains without modifying individual items. Participants completed self‐administered questionnaires; each item was rated on a 5‐point Likert scale (1 = very dissatisfied, 2 = dissatisfied, 3 = somewhat satisfied, 4 = satisfied, 5 = very satisfied) [29], with higher scores indicating greater satisfaction. For each domain, the raw score was calculated as the mean of item scores within that domain; no Rasch transformation was applied.

#### 2.6.2 Time Points and Photographic Pairing

Standardized photographs obtained at three prespecified time points were rated on WSRS and GAIS, according to predefined scoring criteria, by two independent, blinded evaluators. Baseline (pre‐treatment): photographs acquired immediately before the first auricular acupuncture session. Immediate post‐treatment (same day): photographs acquired on the same day after completion of a single auricular acupuncture session, paired with the baseline images for the immediate‐effect assessment. Post‐3‐month (intermediate‐term): photographs acquired after completion of a 3‐month auricular acupuncture treatment course, paired with the baseline images for the intermediate‐term assessment.

At any time point, cases with ineligible or non‐comparable photographs, such as incomplete pre/post pairs, non‐horizontal camera angles, or marked lighting discrepancies, were excluded from the paired analysis for that time point. Analyses at each time point were restricted to participants with complete, comparable paired photographs and complete WSRS, GAIS, and FACE‐Q questionnaire data; no imputation was performed. We report the actual number of participants included in the immediate‐effect and intermediate‐term analyses.

### 2.7 Safety Assessments

Safety assessments encompassed all local and systemic adverse events observed throughout the entire treatment period, including bleeding, bruising, pain, edema, auricular pruritus, infection, and vasovagal syncope. The pain levels were measured using the Visual Analog Scale (VAS), which included four levels: no pain (0 points), mild pain (1–3 points), moderate pain (4–7 points), and severe pain (8–10 points) [30]. During the study, participants were instructed to document any adverse symptoms and their durations. For participants receiving multiple treatments, the condition following the previous treatment was evaluated prior to each subsequent session, and participants were required to provide accurate reports of their symptoms.

### 2.8 Data Analysis

Continuous variables were summarized as mean ± standard deviation (SD), and categorical variables as number (percentage). Within‐participant pre–post comparisons at each time point used paired t‐tests for approximately normally distributed variables and Wilcoxon signed‐rank tests for non‐normally distributed variables. A two‐sided *p* < 0.05 was considered statistically significant.

## 3 Results

### 3.1 Participants Selection and Baseline Characteristics

The screening process identified 242 participants who received auricular acupuncture cosmetic treatment from the outpatient database and written records. After inclusion and exclusion evaluations, the final participant count was recorded. During data collection, 6 participants were excluded for being under 35 years old, and 10 were excluded due to incomplete clinical records, such as missing basic information, lack of documented informed consent, or incomplete questionnaires. During photo review, 9 participants were excluded due to incomplete pre‐ and post‐treatment photos or photos unsuitable for comparison due to issues like angle and lighting. Ultimately, 217 participants met the eligibility criteria and were included in the final analysis. Among these 217 participants, 131 were included in the immediate‐effect analysis and 86 in the intermediate‐term (3‐month) analysis.

The baseline characteristics of the cohort revealed a predominantly female composition, with 95.85% (208 participants) female and only 4.15% (9 participants) male. The participants had a mean age of 48.31 years, ranging from 35 to 70.

The cohort composition, participant selection process, and key baseline characteristics are summarized in Table 1 and Figure S2 for clarity.

**TABLE 1:** Basic information of participants.

Characteristic	No.
Participants	217
Sex	
Male	9 (4.15%)
Female	208 (95.85%)
Age	
Mean (year)	48.31
Range (year)	35–70
35–40 year	47
41–49 year	80
≥ 50 year	90
Treatment session	
1–4	79 (36.40%)
5–8	32 (14.75%)
9–12	20 (9.22%)
> 12	86 (39.63%)

### 3.2 Immediate Effect Assessment

In the comparison of WSRS scores for immediate efficacy, the pre‐treatment mean score was 2.49 ± 0.69, which decreased to 1.86 ± 0.87 post‐treatment (Table 2). The WSRS score decreased in 82 (62.60%) cases and remained unchanged in 49 (37.40%) cases. These findings indicate an improvement in WSRS scores after treatment, with a statistically significant difference (*p* < 0.05).

**TABLE 2:** WSRS of immediate pre‐ and post‐treatment (photo‐based assessment by researchers).

WSRS scale	Pre‐treatment no. (%)	Post‐treatment no. (%)
No wrinkles	None (0.00%)	None (0.00%)
Just perceptible Shallow wrinkles	None (0.00%)	52 (39.70%)
Shallow wrinkles	80 (61.07%)	51 (38.93%)
Moderately deep wrinkles	40 (30.53%)	23 (17.56%)
Deep wrinkles, well‐defined edges	9 (6.87%)	4 (3.05%)
Very deep wrinkles, redundant fold	2 (1.53%)	1 (0.76%)
Mean ± SD	2.49 ± 0.69	1.86 ± 0.87

Abbreviation: SD, standard deviation.

For immediate efficacy assessed using the GAIS, 101 cases (77.10% of the total) were rated as “improved” (scores ≤ 3), indicating a positive aesthetic outcome in the majority of participants (Table 3). Representative cases of immediate effects are illustrated in Figure S3.

**TABLE 3:** GAIS of immediate pre‐ and post‐treatment (photo‐based assessment by researchers).

GAIS scale	Post‐treatment, no. (%)
Very much improved	10 (7.63%)
Greatly improved	51 (38.93%)
Improved	40 (30.54%)
No change	30 (22.90%)
Worse	0 (0.00%)
All improved	101 (77.10%)

### 3.3 Intermediate‐Term (3‐Month) Efficacy Evaluation

In the comparison of WSRS scores for intermediate‐term efficacy, the pre‐treatment score was 2.53 ± 0.66, which decreased to 1.67 ± 0.73 after 3 months of treatment. Among the participants, 67 (77.91%) showed a reduction in scores, 19 (22.09%) had unchanged scores (Table 4). The statistical analysis revealed a significant improvement in WSRS scores after treatment (*p* < 0.05).

**TABLE 4:** WSRS of intermediate‐term pre‐ and post‐treatment (photo‐based assessment by researchers).

WSRS scale	Pre‐treatment no. (%)	Post‐3 months, no. (%)
No wrinkles	None (0.00%)	None (0.00%)
Just perceptible Shallow wrinkles	None (0.00%)	40 (46.51%)
Shallow wrinkles	48 (55.81%)	35 (40.70%)
Moderately deep wrinkles	30 (34.89%)	10 (11.63%)
Deep wrinkles, well‐defined edges	8 (9.30%)	1 (1.16%)
Very deep wrinkles, redundant fold	None (0.00%)	None (0.00%)
Mean ± SD	2.53 ± 0.66	1.67 ± 0.73

For intermediate‐term efficacy evaluated by GAIS, 82 participants (representing 95.34% of the total) were rated as improved (scores ≤ 3) (Table 5). Representative cases of intermediate‐term effects are shown in Figure S4.

**TABLE 5:** GAIS of intermediate‐term pre‐ and post‐treatment (photo‐based assessment by researchers).

GAIS scale	Post‐treatment, no. (%)
Very much improved	19 (22.09%)
Greatly improved	36 (41.86%)
Improved	27 (31.40%)
No change	4 (4.65%)
Worse	0 (0.00%)
All improved	82 (95.35%)

### 3.4 Participant Satisfaction Evaluation

The results regarding participant satisfaction indicated that immediately after auricular acupuncture treatment, the satisfaction with overall facial appearance was 3.36 ± 0.76, satisfaction with improvement in the cheek area was 3.54 ± 0.65, the improvement in the lower face and jawline was 3.35 ± 0.72, and the improvement in nasolabial folds was 3.59 ± 0.62 (*p* < 0.05). The results of the intermediate‐term auricular acupuncture treatment showed that the satisfaction with overall facial appearance was 3.63 ± 0.72, the improvement in the cheek area was 3.78 ± 0.64, the improvement in the lower face and jawline was 3.58 ± 0.77, and the improvement in nasolabial folds was 3.77 ± 0.68 (*p* < 0.05; Table 6).

**TABLE 6:** FACE‐Q Scale: Immediate and intermediate‐term satisfaction with facial appearance.

	Immediate post‐treatment	Intermediate‐term post‐treatment
	Mean ± SD (*N* = 131)	Mean ± SD (*N* = 86)
FACE‐Q scale (range 1 to 5)		
Satisfaction with overall facial appearance	3.36 ± 0.76	3.63 ± 0.72
Satisfaction with cheeks	3.54 ± 0.65	3.78 ± 0.64
Satisfaction with lower face and Jawline	3.35 ± 0.72	3.58 ± 0.77
Improvement of nasolabial folds	3.59 ± 0.62	3.77 ± 0.68

Overall, the immediate effect on nasolabial folds received the highest satisfaction score, indicating that participants were most satisfied with improvement in this area immediately after treatment. After the 3‐month treatment course, the highest satisfaction score was observed for the cheeks, suggesting that participants were particularly pleased with cheek firmness following prolonged treatment. Across all FACE‐Q domains, mean scores were above 3.0 (somewhat satisfied) at both time points, with numerically higher scores after the 3‐month course than after a single treatment session.

### 3.5 Safety Evaluation

Most participants reported only mild pain at the needle insertion site. According to the VAS scores, 76 participants (35.02%) reported no pain, while the remaining 141 participants (64.98%) reported mild pain, predominantly scoring 1–2. These findings indicate a low level of discomfort associated with auricular acupuncture, demonstrating good tolerance among participants, which facilitates positive compliance with the treatment. Seventy‐nine participants experienced bleeding after needle insertion, which was successfully controlled with pressure from a dry cotton swab for 1–2 min. One participant experienced faintness after acupuncture, presenting with pallor, nausea, and sweating. After the needles were removed immediately and the participant rested in bed for about 20 min, the symptoms resolved. It is noteworthy that no severe adverse effects, such as intense pain or infection, were observed in any participants (Table 7).

**TABLE 7:** Distribution of adverse events among participants.

Adverse event or adverse effect	Participants, no. (%)
Mild and transient adverse effects	
Mild pain (VAS: 1–3)	141 (64.98%)
Intense pain (VAS: 4–10)	0 (0.00%)
Bleeding	79 (36.41%)
Bruising	2 (0.92%)
Edema	1 (0.46%)
Auricular pruritus	1 (0.46%)
Vasovagal syncope	1 (0.46%)
Moderate to severe adverse events	
Infection	0 (0.00%)

## 4 Discussion

This study is the first to evaluate the cosmetic efficacy of auricular acupuncture using a standardized rating‐scale system. Our retrospective analysis suggests that auricular acupuncture may produce immediate and intermediate‐term improvements in facial aesthetic outcomes, as evidenced by post‐treatment reductions in WSRS scores. The technique is characterized by relatively rapid onset, minimal invasiveness, a simple two‐needle protocol, and ease of mastery, making it a potentially feasible option in clinical aesthetic practice. In this study, the immediate improvements in facial contour and jawline definition were associated with a GAIS response rate of 77.10%, and the 3‐month treatment indicated a certain degree of persistence, with a GAIS effectiveness rate of 95.35%.

From November 2023 to December 2024, we included 242 patients who received auricular acupuncture for aesthetic purposes at our institution. Although a large number of cosmetic consultations were conducted over the past year, this retrospective study enrolled only participants who met strict inclusion criteria, exhibited evident signs of aging, and had complete data. Notably, some patients with apparently favorable responses declined consent for the use of pre‐ and post‐treatment photographs due to privacy concerns or other personal reasons, leading to their exclusion from the imaging‐based analyses. In addition, some patients did not complete the 3‐month follow‐up, which limited the sample size for intermediate‐term evaluation. Accordingly, the intermediate‐term findings should be regarded as suggestive and warrant confirmation in larger prospective cohorts. We excluded participants seeking aesthetic treatment without obvious wrinkles; 16 participants were removed for incomplete data, and 9 more were excluded at image review due to non‐level camera angles or insufficient depiction of aging signs. Although missingness was deemed random, attrition occurred. To support consistency, we used standardized photography to align lighting, angle, and expression. During evaluation, raters were independent and blinded to clinical information, but the pre/post nature of paired images could not be fully masked. To mitigate expectation bias, we used predefined scoring criteria, dual independent ratings, and adjudication of discrepancies.

The key techniques in this trial involve accurate localization and precise operation. Given the numerous ear acupuncture points and the small size of the ear, accuracy in locating the points is crucial for successful treatment. The positions of the three key points can be determined using the relevant anatomical landmarks of the ear. Additionally, the needling technique is vital. Specific techniques should be employed during the procedure, and after inserting the needles, the practitioner needs to observe the immediate changes. If no noticeable changes occur, the practitioner can use a twisting technique by rotating the needle handle, typically within a range of 90 to 180 degrees. This maneuver can enhance the stimulation of the acupuncture points, thereby improving the therapeutic effect. It is worth noting that we recommend participants leave the needles in place for at least 2 h. Based on our observations, the longer the needles are retained, the better the treatment effect is maintained. Therefore, we extended the needle retention time from the traditional 20 to 30 min to a longer duration. This approach also takes into account the convenience of auricular acupuncture, as leaving the needles in the ear does not significantly affect the participant's daily life and work.

The mechanism underlying auricular acupuncture for aesthetic purposes remains unclear [31]. The neurological theory suggests that acupuncture stimulates nerve branches in the auricle, transmitting signals to the brain, which are then relayed through the spinal cord to corresponding regions, producing therapeutic effects [32]. The acupuncture points selected in this study are near the auriculotemporal nerve, with the anterior branch innervating the parotid gland and mandibular angle, and the posterior branch innervating the mastoid process and posterior auricle [33]. Dr. Nogier's embryological theory proposes that the auricle develops from tissue along the first pharyngeal arch [34]. The selected acupuncture points lie in the ectoderm of this arch, which provides innervation to facial structures [35]. The biological holography theory suggests that acupuncture points in the cheek area are effective for facial treatment [21].

Current evidence on auricular acupuncture for reducing facial wrinkles and enhancing facial contour remains limited. Most published studies have instead focused on techniques such as laser therapy, thread lifting, injectable fillers, or facelift surgery, whose therapeutic targets, candidate populations, and follow‐up durations are not fully comparable to those of auricular acupuncture [36, 37, 38]. By contrast, auricular acupuncture may offer a less invasive, more convenient option with faster recovery for participants seeking aesthetic treatments who are particularly sensitive to procedural trauma or who require higher tolerability. In addition, the growing number of reports on auricular acupuncture for conditions such as melasma, urticaria, and acne further supports its potential role as an adjunctive therapy or an interval maintenance approach alongside other cosmetic or dermatologic treatments [39].

## 5 Limitations

Firstly, this was a retrospective, single‐center observational study without a control group, which limits causal inference and makes it difficult to fully exclude confounding and placebo effects. Secondly, the study was not prospectively designed around a single primary endpoint, and no a priori sample‐size calculation was performed, which may limit the precision of estimates and the generalizability of the findings. Thirdly, outcome assessment relied on subjective scales and photo‐based ratings; despite standardized photography, blinded dual rating, and predefined criteria, residual measurement error may persist due to subtle differences in lighting, posture, and facial expression. In addition, the abbreviated FACE‐Q subset used in this cohort was not psychometrically validated, reducing cross‐study comparability. Fourthly, the treatment period was approximately 3 months, which is insufficient to evaluate durability of the effect, and longer‐term follow‐up outcomes were not available. Fifthly, the sample was predominantly female and there was attrition related to incomplete information or imaging data, so extrapolation, especially to male patients, should be made cautiously and ideally verified in cohorts with more balanced sex representation. Sixthly, the study did not include objective instrumental measures such as three‐dimensional facial imaging, skin elasticity testing with a Cutometer, high‐frequency ultrasound, or standardized cross‐polarized photography.

## 6 Conclusions

This study suggests that auricular acupuncture has significant effects on both immediate and intermediate‐term (3‐month) facial enhancement. Immediate effects primarily included facial tightening and lifting, while intermediate‐term effects (observed over 3 months) also encompassed wrinkle reduction, enhanced rejuvenation, and increased patient satisfaction. Thus, this retrospective study provides preliminary evidence supporting the role of auricular acupuncture in facial aesthetics. Nonetheless, in view of multiple inherent limitations as a retrospective study, the findings should be regarded as preliminary and interpreted with caution. Future studies should employ prospective, adequately powered multicenter designs with longer follow‐up, validated patient‐reported outcomes, standardized image acquisition, and integration of objective imaging and biomechanical metrics.

## Author Contributions

W.Z. and P.P. drafted the initial manuscript and were responsible for data integrity and analysis. Y.H., H.H., and L.C. critically reviewed and revised the overall content. All authors participated in data collection, data analysis, and reviewed and approved the final version.

## Funding

The authors have nothing to report.

## Conflicts of Interest

The authors declare no conflicts of interest.

## Supporting information


**Data S1:** jocd70629‐sup‐0001‐Figures.docx.

## Data Availability

The datasets supporting the findings of this study are available from the corresponding author upon reasonable request. Due to privacy and ethical restrictions, these data are not publicly available.
