# Supplementary material for: Auricular Acupuncture for Facial Aesthetics: A Preliminary Retrospective Clinical Study of 217 Cases
Source: J Cosmet Dermatol. 2026 Jan 7;25(1):e70629. doi: 10.1111/jocd.70629 (PMC12779092; doi:10.1111/jocd.70629)
Supplement: Supplementary file 1 — Data S1: jocd70629‐sup‐0001‐Figures.docx. [file JOCD-25-e70629-s001.docx]

**Supplementary Material**


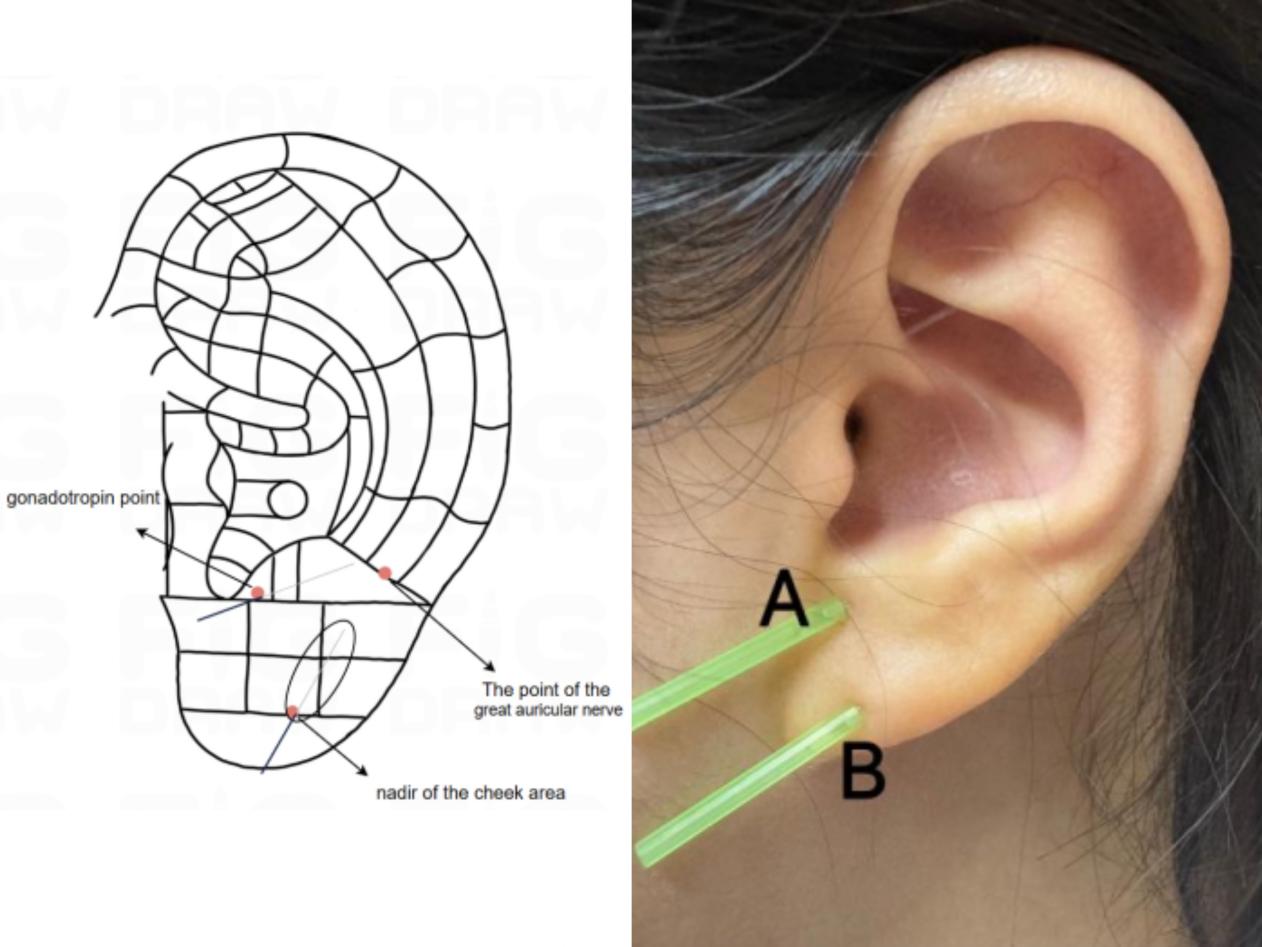


Supplementary Figure S 1. Location of the three selected auricular point points(self-drawn, adapted from GB/T13734-2008)

*Schematic diagram of auricular acupuncture.Note:The gonadotropin (A) and the lowest point of the cheek area (B) are selected and the needle tip is inserted towards the direction of the major auricular nerve point. The needle body is completely inserted and left in the fat layer.*


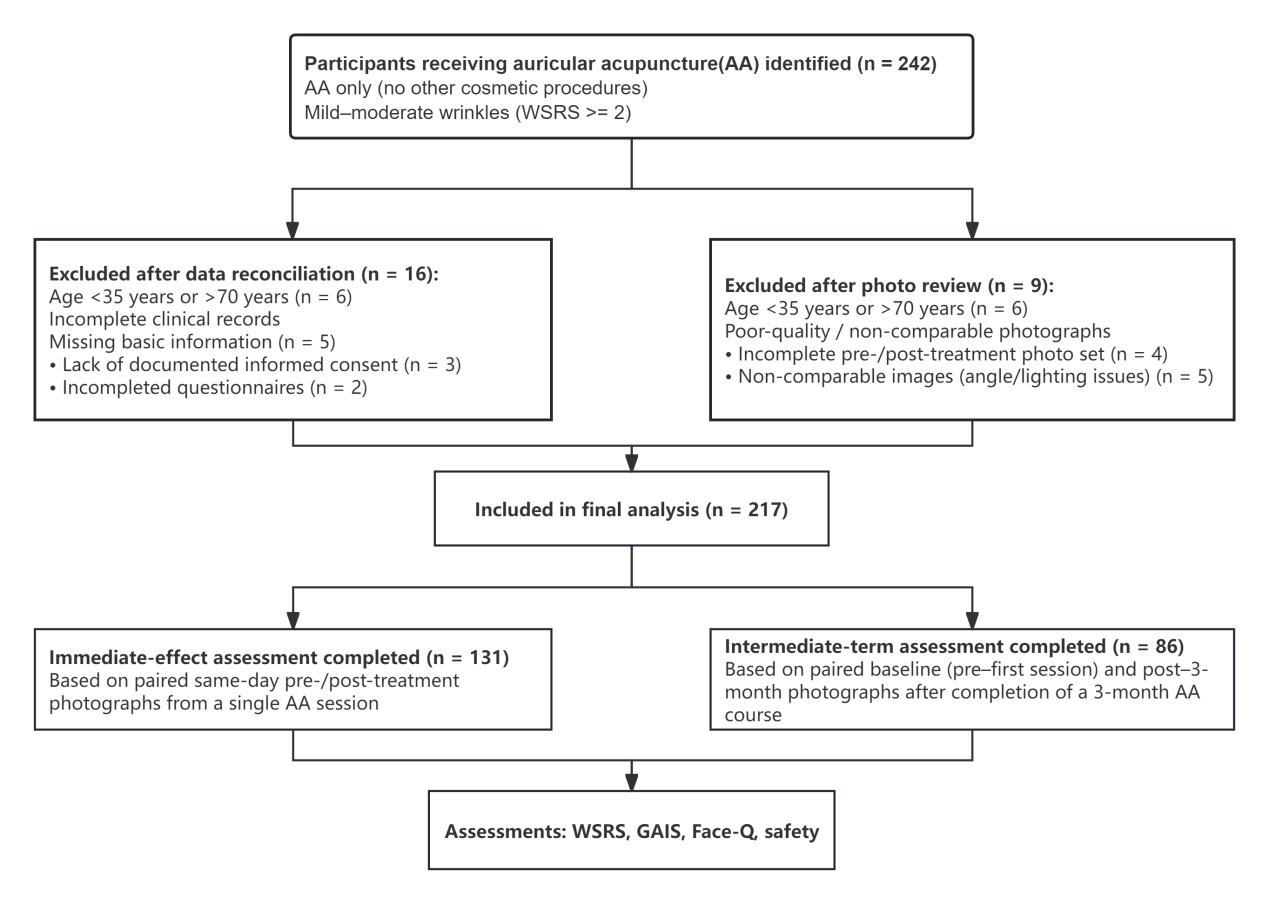


Supplementary Figure S 2. **Flowchart of Screening and Follow-Up.**

**
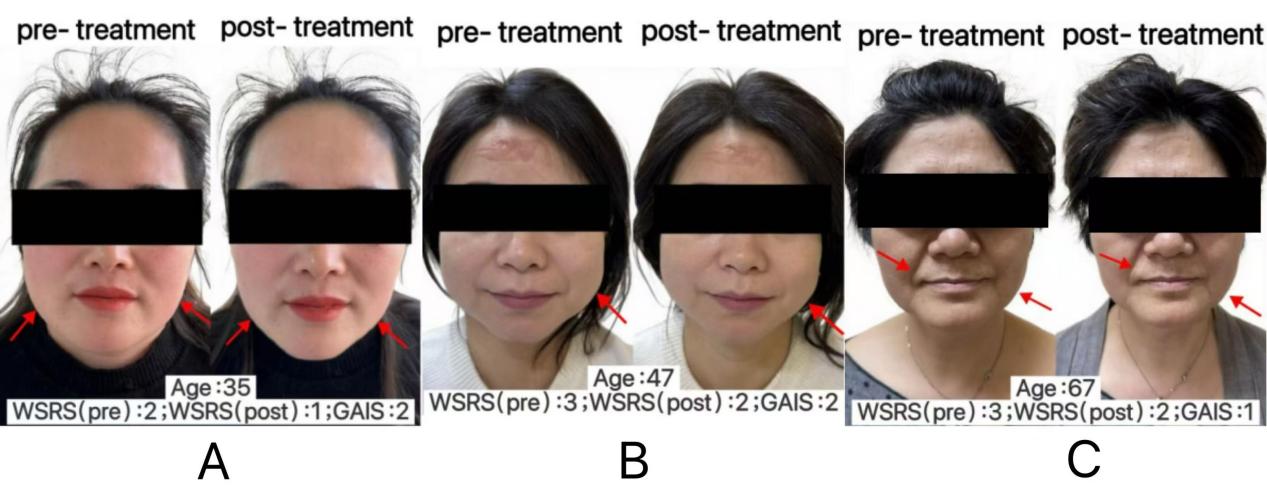
**

Supplementary Figure S 3. **Representative cases of immediate effects.**

*(A)Case 1, after treatment, the patient showed bilateral cheek tightening and a more youthful face. (B) Case 2, before treatment, she showed significant facial asymmetry, with the left side of her face larger than the right. After treatment, the patient's facial asymmetry improved. (C) Case 3, after treatment, the participant showed a reduction of the right side of the striae and a tightening of the cheek on the left side of the face.*

**
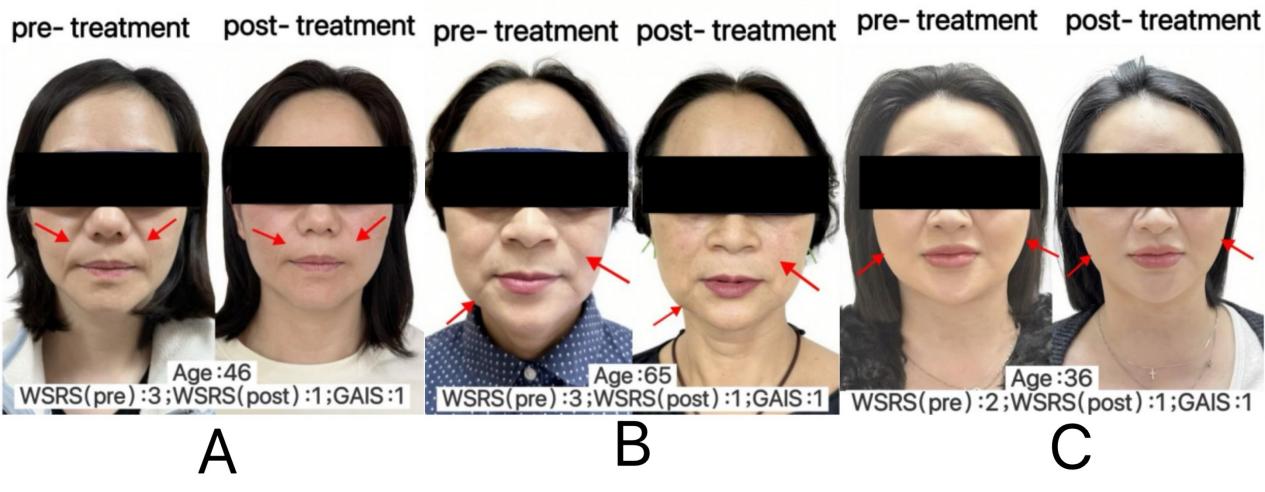
**

Supplementary Figure S 4. Representative cases of intermediate-term effects.

*(A) Case 1, the participant's wrinkles were significantly reduced, the facial skin was more shiny, the overall face was smooth, and the face was more youthful. (B) Case 2, after treatment, the facial lines and fine lines were relieved, the facial contours were tightened, and the mouth pouches were loosened. (C) Case 3, three months after treatment, the participant showed tightness in both sides of the cheeks, more fluid alignment of the jawline, and significant improvements in the size of the face.*
